# Structure refinement of (NH_4_)_3_Al_2_(PO_4_)_3_ prepared by ionothermal synthesis in phospho­nium based ionic liquids – a redetermination

**DOI:** 10.1107/S2056989019015330

**Published:** 2019-11-19

**Authors:** Christopher P. Nicholas, John P.S. Mowat, Robert W. Broach

**Affiliations:** a Exploratory Materials and Catalysis Research, Honeywell UOP, Des Plaines IL 60201, USA; b Advanced Characterization, Honeywell UOP, Des Plaines IL 60201, USA

**Keywords:** powder diffraction, aluminophosphate, ionothermal synthesis, ethyl­tri­butyl­phospho­nium di­ethyl­phosphate, Cyphos 169, redetermination

## Abstract

After crystallization during ionothermal syntheses in phospho­nium-containing ionic liquids, the structure of (NH_4_)_3_Al_2_(PO_4_)_3_ [tri­ammonium dialuminum tris­(phosphate)] was refined on the basis of powder X-ray diffraction data from a synchrotron source. (NH_4_)_3_Al_2_(PO_4_)_3_ is a member of the structural family with formula *A*_3_Al_2_(PO_4_)_3_, where *A* is a group 1 element, and of which the NH_4_, K, and Rb forms were previously known. The NH_4_ form is isostructural with the K form, and was previously solved from single-crystal X-ray data when the material (SIZ-2) crystallized from a choline-containing eutectic mixture [Cooper *et al.* (2004 ▸). *Nature*, **430**, 1012–1017]. Our independent refinement incorporates NH_4_ groups and shows that these NH_4_ groups are hydrogen bonded to framework O atoms present in rings containing 12 *T* sites in a channel along the *c-*axis direction. We describe structural details of (NH_4_)_3_Al_2_(PO_4_)_3_ and discuss differences with respect to isostructural forms.

## Abstract 2

The crystal structure of (NH_4_)_3_Al_2_(PO_4_)_3_ was refined by powder XRD synchrotron data. (NH_4_)_3_Al_2_(PO_4_)_3_ is a member of the structural family with formula *A*_3_Al_2_(PO_4_)_3_ where *A* is a group 1 element, of which the K and Rb forms are also known.

## Chemical context   

Following the discovery of the microporous AlPO_4_-*n* series of materials (Wilson *et al.*, 1982 ▸), many efforts have been directed toward the synthesis of novel phases utilizing traditional hydro­thermal (Wilson, 2007 ▸; Yu & Xu, 2006 ▸) and solvothermal syntheses (Das *et al.*, 2012 ▸). Recently, ionothermal synthesis has been added to the stable of synthetic methods. Ionothermal synthesis is an extension of the solvothermal method of synthesis using an ionic liquid as the solvent (replacing, for example, water or ethyl­ene glycol) where a portion of the organic structure-directing agent from a typical zeolite synthesis is derived from the ionic liquid (Morris, 2009 ▸). Many new materials have been synthesized by ionothermal synthesis, with new aluminophosphate materials among the most common (Parnham & Morris, 2007 ▸; Xing *et al.*, 2008 ▸, 2010 ▸).

An important issue in ionothermal synthesis is control of water (Ma *et al.*, 2008 ▸). Excess water often leads to synthesis of dense AlPO_4_ phases such as the one with a tridymite-type of structure, which we observed as well during syntheses utilizing 85%_wt_ H_3_PO_4_. To control the level of water in the synthesis, thereby allowing easy recycling of the ionic liquid solvent and to intentionally prepare ammonium aluminophosphates, we used (NH_4_)_2_HPO_4_ as the phospho­rous source in the synthesis. Ammonium is a good structure-directing agent for aluminophosphate frameworks; multiple ammonium aluminum phosphates are known (Byrne *et al.*, 2009 ▸; Vaughan *et al.*, 2012 ▸). In the current phospho­nium-based ionothermal synthesis, the presence of an ammonium cation in the relative absence of water provokes the formation of a 2/3 Al/P framework with the formula (NH_4_)_3_Al_2_(PO_4_)_3_. A structurally unrelated compound with the formula (NH_4_)_3_Al_2_(PO_4_)_3_ has previously been synthesized *via* a solvothermal approach (Medina *et al.*, 2004 ▸).

The aluminophosphate database at Jilin (Li *et al.*, 2019 ▸) currently lists 21 framework structures with a 2:3 ratio of Al:P. A framework with sub-stoichiometric Al content is by necessity anionically charged and must be cation-balanced, so most of the known frameworks, such as UT-3, UT-4 and UT-5 (Oliver *et al.*, 1996 ▸) are charge-balanced by organo­ammonium cations. Low-water-content syntheses clearly favor 2:3 compounds as most of the known materials are synthesized from low-water-content preparations.

## Structural commentary and survey of related compounds   

**Figure 1:**
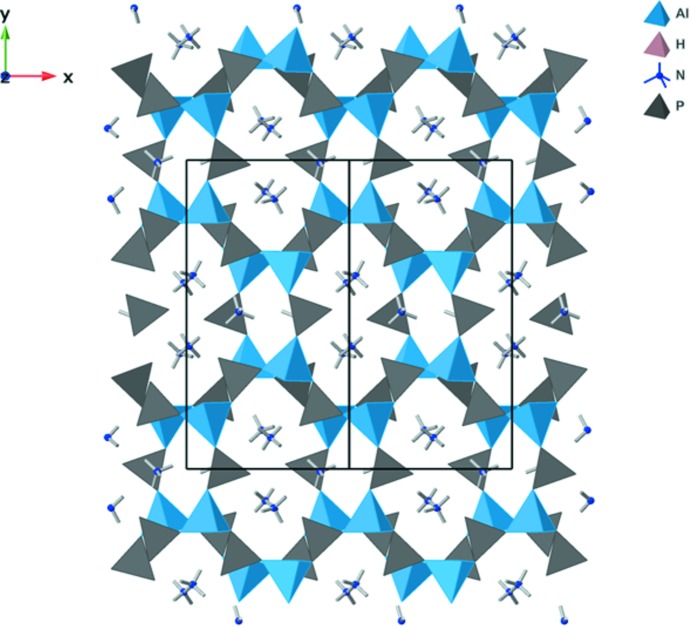
Polyhedral representation of (NH_4_)_3_Al_2_(PO_4_)_3_, showing the overall connectivity and ion channels in the crystal structure. Al is in the center of blue tetra­hedra, P in gray tetra­hedra, and N is represented by blue spheres.

The (NH_4_)_3_Al_2_(PO_4_)_3_ phase synthesized here is related to the series of *A*_3_Al_2_(PO_4_)_3_ materials synthesized *via* high-temperature solid-state methods (Devi & Vidyasagar, 2000 ▸) with varying monocations on the *A* site. Additionally, an independent synthesis previously yielded a (NH_4_)_3_Al_2_(PO_4_)_3_ material called SIZ-2 whose structure was solved and refined from single-crystal data (Cooper *et al.*, 2004 ▸) and possesses nearly the same structure as refined from the current powder data of (NH_4_)_3_Al_2_(PO_4_)_3_. A polyhedral representation of the crystal structure of (NH_4_)_3_Al_2_(PO_4_)_3_ is shown in Fig. 1 ▸. SIZ-2 crystallized from a choline chloride/urea eutectic mixture where decomposition of urea was proposed to be the source of ammonium in the structure. The refinement of Cooper *et al.* (2004 ▸) included the ammonium N atoms, but made no attempt to find or model the corresponding H atoms.

Devi & Vidyasagar (2000 ▸) utilized Li, Na, K, Rb, Cs, and Tl as the *A* cation and succeeded in crystallizing compounds with *A* = Na, K, Rb, Tl. The thallium derivative yielded a completely different structure with trigonal–bipyramidal coordination of Al. The *A* = Na structure was not solved, but apparently crystallizes in an unrelated ortho­rhom­bic space-group type from that observed for *A* = K, Rb in their work, and for *A* = NH_4_ here. Devi & Vidyasagar (2000 ▸) utilized (NH_4_)_2_HPO_4_ as the phosphate source in their high-temperature preparations of *A*_3_Al_2_(PO_4_)_3_, but did not obtain (NH_4_)_3_Al_2_(PO_4_)_3_, likely due to the volatility of NH_3_ at high temperatures.

**Figure 2:**
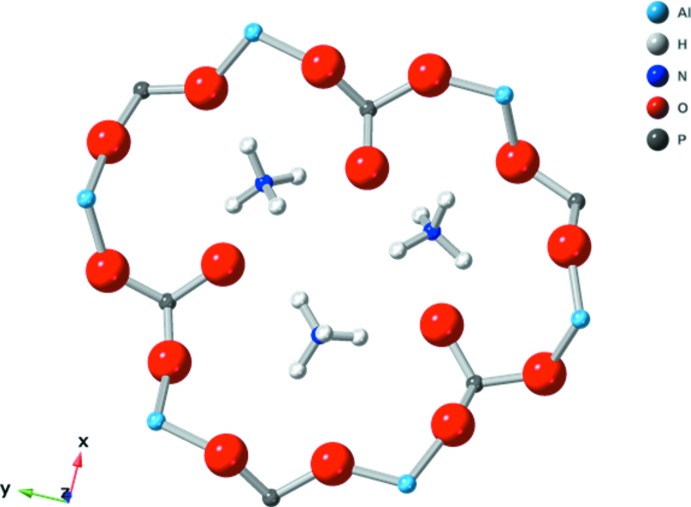
Ball and stick representation of (NH_4_)_3_Al_2_(PO_4_)_3_ showing the 12-membered ring with three phosphate groups protruding inward with close contact to ammonium cations.

As in the K and Rb forms of the *A*_3_Al_2_(PO_4_)_3_ series, aluminum and phospho­rus are both tetra­hedrally coordinated and connected through corners throughout the (NH_4_)_3_Al_2_(PO_4_)_3_ structure. The NH_4_^+^ cations reside in a channel along the *c-*axis direction made from a 12 *T*-site ring of alternating AlO_4_ and PO_4_ tetra­hedra (Fig. 2 ▸). The NH_4_^+^ groups occupy the available space and none of the ionic liquid solvent is present within the pores of the (NH_4_)_3_Al_2_(PO_4_)_3_ framework. Without the NH_4_^+^ groups, the structure would have 24% void volume. The framework is triply negatively charged and charge-balanced by the ammonium cations. Three of the six phosphate groups in the ring protrude inward such that the closest contact distance between the H atom of an ammonium group and the O atom of the nearest phosphate is between 1.83 and 1.87 Å, indicating significant hydrogen-bonding inter­actions. The full range of H⋯O hydrogen-bond lengths is between 1.83 and 1.97 Å (Table 1 ▸).

Crystallizing in space-group type *Pna*2_1_, (NH_4_)_3_Al_2_(PO_4_)_3_ is isostructural to, but with a slightly larger unit cell than the K form synthesized by Devi & Vidyasagar (2000 ▸). Lattice expansion of ∼0.1–0.2 Å occurs along each of the three axes, leading to an overall 6.6% increase in cell volume from 1245 to 1327 Å^3^. A lattice expansion is no surprise as the ionic radius of NH_4_^+^ is between 1.4 and 1.67 Å depending on the coordination number (Sidey, 2016 ▸). This is slightly larger than the reported 1.37 to 1.55 Å range for K^+^ (Shannon, 1976 ▸). Much of the relative lattice expansion for (NH_4_)_3_Al_2_(PO_4_)_3_ occurs along the *a* and *c* axes. Tilting of tetra­hedra accounts for a significantly smaller expansion of the long *b* axis. In addition, an isostructural K/As form is also known where two-thirds of the phosphate groups have been replaced by arsenate (Boughzala *et al.*, 1997 ▸). Arsenate included on the phosphate sites increases the cell volume to 1307 Å^3^, just smaller than that recorded here for (NH_4_)_3_Al_2_(PO_4_)_3_. The pure arsenate form K_3_Al_2_(AsO_4_)_3_ was reported by Stöger & Weil (2012 ▸), which has a cell volume of 1328 Å^3^, essentially equivalent to that here.

**Figure 3:**
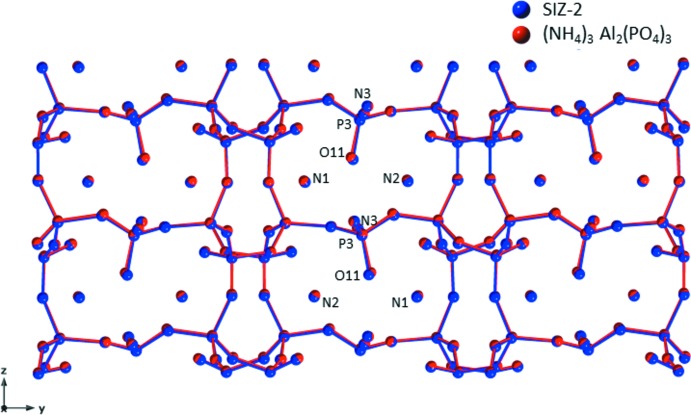
Atomic position overlay plot of SIZ-2 (blue) and (NH_4_)_3_Al_2_(PO_4_)_3_ (red) showing that most atom positions are within 0.03 Å of each other. The most significant difference is in the O11 position.

**Figure 4:**
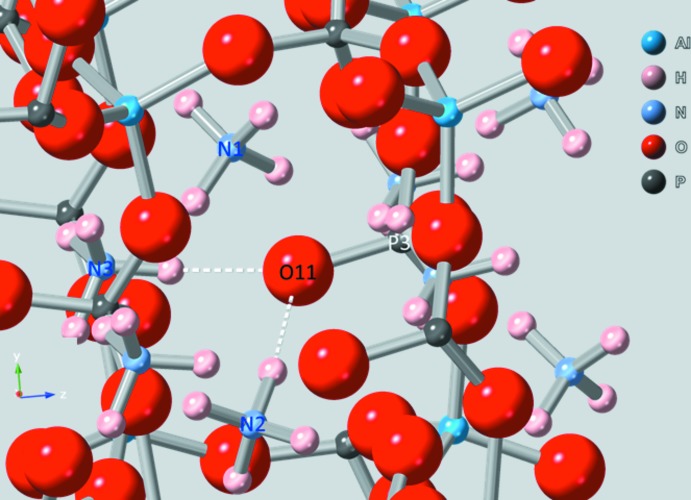
Ball and stick representation of the key area surrounding O11 where the largest position movement takes place in the two independent refinements of (NH_4_)_3_Al_2_(PO_4_)_3_.

An overlay plot of atomic positions of (NH_4_)_3_Al_2_(PO_4_)_3_ (red) *versus* SIZ-2 (blue) shows that although the independent refinements of the two (NH_4_)_3_Al_2_(PO_4_)_3_ materials were performed *via* different methods at different temperatures, most atom positions are similar, with no more than about 0.004 fractional position differences along the *a* or *c* axes (for these axes, about 0.03–0.04 Å, Fig. 3 ▸). One area stands out in the *A*_3_Al_2_(PO_4_)_3_ series. Fig. 4 ▸ shows the key area surrounding O11 where the largest position movement is observed in the two independent refinements of (NH_4_)_3_Al_2_(PO_4_)_3_.

The P3—O11 bond is always among the shortest P—O bonds found in the crystal structure, here at 1.487 (5) Å. Two clusters of P—O bond lengths occur; one at about 1.49 Å and another at 1.55 Å. These distances are relatively typical for aluminophosphates (Richardson & Vogt, 1992 ▸; Wei *et al.*, 2012 ▸). Each of the O atoms protruding into the pore possess short P—O bonds and hydrogen bonds to two ammonium ions (Table 1 ▸). In particular, N2, N3, O11, and P3 are effectively in a plane so that with the hydrogen bonding present in our refined model from N3 and N2 through the attached H atoms to O11, O11 moves closer to P3 while N2 and N3 move slightly further away *versus* the positions in the SIZ-2 refinement. Table 2 ▸ shows respective O—*A* and P—O distances for the four isostructural *A*_3_Al_2_(PO_4_)_3_ compounds. Other bond lengths and angles are otherwise relatively unremarkable *versus* other members of the structural class although we note that As/P—O distances are longer than P—O as expected.

Rb_3_Al_2_(PO_4_)_3_ is structurally related to the NH_4_ and K forms, but crystallizes in a higher symmetry space-group type (*Cmc*2_1_), accompanied with higher overall coordination numbers around Rb^+^ and a mirror plane perpendicular to *a*. The ionic radius of Rb^+^ is similar to that of NH_4_^+^, reported as 1.52–1.63 Å (Shannon, 1976 ▸). Lithium and cesium forms of the series have not yet been synthesized, likely because of the relatively small and large, respectively, ionic radii *versus* those of the fitting *A* cations. Our initial attempts at ion-exchange of (NH_4_)_3_Al_2_(PO_4_)_3_ with LiNO_3_ or CsNO_3_ in aqueous solution to form the Li or Cs form failed, with partial structural degradation and no ion-exchange observed.

## Synthesis and crystallization   

**Figure 5:**
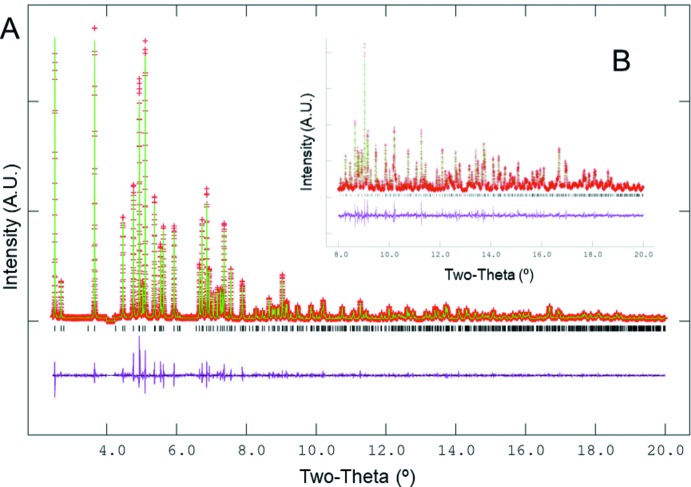
XRD pattern (*λ* = 0.373811 Å) of (NH_4_)_3_Al_2_(PO_4_)_3_ synthesized ionothermally in ethyl tri­butyl­phospho­nium di­ethyl­phosphate and Rietveld residuals following structure refinement. Part A shows the fit to the overall pattern, and inset B shows the fit to high-angle regions.

**Figure 6:**
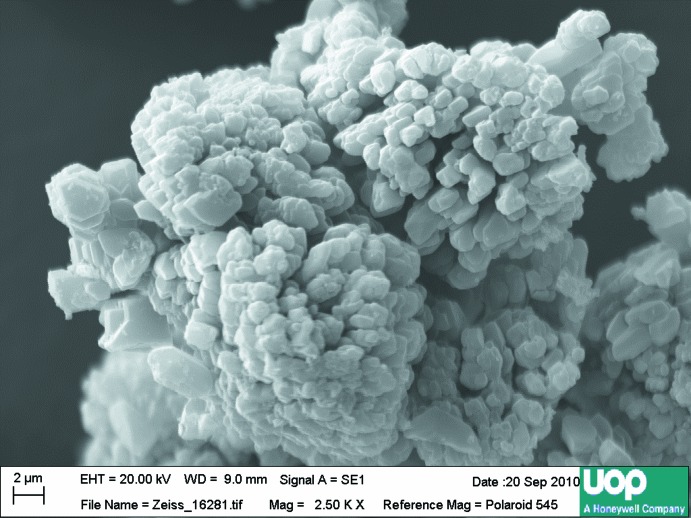
SEM image of polycrystalline (NH_4_)_3_Al_2_(PO_4_)_3_ synthesized ionothermally in ethyl tri­butyl­phospho­nium diethyl phosphate and used for structure refinement.

In a typical preparation, 1.65 g (NH_4_)_2_HPO_4_ was added to a 125 ml polytetra­fluoro­ethene (PTFE) lined autoclave containing 24.02 g of ethyl tri(but­yl)phospho­nium di­ethyl phosphate. The mixture was stirred at room temperature for 2 min. To this mixture were added 0.49 g of Al(OH)_3_, and the contents were stirred at room temperature for 2 min. The contents of the autoclave were digested at 423 K for 24 h prior to isolating the product by filtration. Analytical results show this material has a molar ratio Al:P of 0.725. The X-ray diffraction pattern is shown in Fig. 5 ▸. Scanning electron microscopy (SEM) revealed agglomerated stacks of irregularly shaped blocky crystals of from 500 nm to 2–4 µm in length (Fig. 6 ▸). Calcination of (NH_4_)_3_Al_2_(PO_4_)_3_ at temperatures of 773 K or higher causes the formation of an AlPO_4_ phase with a tridymite-type structure. Ethyl tributyl phospho­nium diethyl phosphate (Cyphos 169) was acquired from Cytec; aluminum hydroxide was acquired from Pfaltz and Bauer.

## Refinement   

Crystal data, data collection and structure refinement details are summarized in Table 3 ▸. Following initial survey scans on in-house Cu source powder XRD instruments, final data were acquired from samples packed in thin glass capillaries on 11-BM at the Advanced Photon Source at Argonne National Laboratory. Starting atomic positions for the refinement were adapted from the literature examples. Starting positions for the ammonium cations were located in a difference-Fourier map and subsequently refined using *GSAS* (Larson & Von Dreele, 2000 ▸) as tetra­hedral rigid bodies with N—H bond lengths held at 0.9526 Å and tetra­hedrality enforced, leading to H⋯H distances of 1.5556 Å. No soft constraints were applied to the framework positions. All atoms in the structure were refined with a common *U*_iso_ parameter. Two low-intensity reflections in the region 4.00–4.22°/2*θ* were excluded from the refinement as belonging to an impurity phase after assessment of multiple (NH_4_)_3_Al_2_(PO_4_)_3_ batches. Refinement trials with a higher symmetry model (space-group type *Cmc*2_1_) were attempted but showed poor agreement with the experimental data, with *R*_wp_ > 0.16.

## Supplementary Material

Crystal structure: contains datablock(s) I. DOI: 10.1107/S2056989019015330/wm5528sup1.cif

CCDC references: 1965580, 1965580

Additional supporting information:  crystallographic information; 3D view; checkCIF report

## Figures and Tables

**Table 1:** Hydrogen-bond geometry (Å, °)

*D*—H⋯*A*	*D*—H	H⋯*A*	*D*⋯*A*	*D*—H⋯*A*
N1—H11⋯O9^i^	0.95 (4)	2.39 (4)	3.250 (10)	151 (3)
N1—H11⋯O11	0.95 (4)	2.35 (5)	3.163 (8)	143 (3)
N1—H12⋯O1^ii^	0.95 (4)	1.88 (4)	2.791 (10)	159 (3)
N1—H13⋯O5	0.95 (4)	2.14 (4)	2.934 (10)	141 (3)
N1—H14⋯O9^iii^	0.95 (4)	1.83 (4)	2.776 (9)	173 (3)
N2—H21⋯O5^iv^	0.95 (4)	1.96 (4)	2.896 (10)	170 (4)
N2—H22⋯O8^v^	0.95 (4)	2.31 (3)	3.216 (10)	158 (4)
N2—H23⋯O9^iv^	0.95 (4)	1.89 (5)	2.738 (9)	148 (4)
N2—H24⋯O11^vi^	0.96 (4)	1.86 (4)	2.818 (9)	174 (5)
N3—H31⋯O5^vi^	0.96 (4)	1.97 (4)	2.821 (9)	147 (3)
N3—H32⋯O11^vi^	0.952 (15)	1.85 (2)	2.728 (8)	153 (4)
N3—H33⋯O1^v^	0.95 (3)	1.90 (3)	2.823 (9)	164 (4)
N3—H34⋯O12	0.96 (3)	2.37 (4)	2.925 (8)	117 (4)

Symmetry codes: (i) 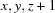; (ii) 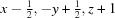; (iii) 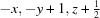; (iv) 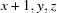; (v) 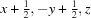; (vi) 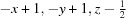.

**Table 2:** Key atomic distances (Å) in related *A*_3_Al_2_(PO_4_)_3_ structures

Compound	O11—*A*1	O11—*A*2	O11—*A*3	O11—P3	Reference
(NH_4_)_3_Al_2_(PO_4_)_3_	3.162	2.818	2.727	1.487	This work
SIZ-2 (*A* = NH_4_)	3.090	2.834	2.688	1.496	Cooper *et al.* (2004 ▸)
K_3_Al_2_(PO_4_)_3_	2.754	2.824	2.722	1.487	Devi & Vidyasagar (2000 ▸)
K_3_Al_2_(AsO_4_)_2_(PO_4_)	3.025	2.743	2.621	1.673	Boughzala *et al.* (1997 ▸)

For each of the compounds, the atomic numbering scheme of the current (NH_4_)_3_Al_2_(PO_4_)_3_ refinement has been utilized. For the first two compounds, *A* = NH_4_, while for the second two, *A* = K. For the As-containing compound, the P3 site is reported to have the highest occupancy of As at 0.86.

**Table 3:** Experimental details

Crystal data	
Chemical formula	(NH_4_)_3_Al_2_(PO_4_)_3_
*M*_r_	392.99
Crystal system, space group	Orthorhombic, *P* *n* *a*2_1_
Temperature (K)	100
*a*, *b*, *c* (Å)	8.98884 (6), 17.01605 (10), 8.67653 (5)
*V* (Å^3^)	1327.11 (2)
*Z*	4
Radiation type	Synchrotron, λ = 0.373811 Å
μ (mm^−1^)	0.12
Specimen shape, size (mm)	Cylinder, 0.70 × 0.70

Data collection	
Diffractometer	11BM synchrotron
Specimen mounting	Capillary
Data collection mode	Transmission
Scan method	Continuous
2θ values (°)	2θ_min_ = 2.45, 2θ_max_ = 20, 2θ_step_ = 0.001

Refinement	
*R* factors and goodness of fit	*R*_p_ = 0.082, *R*_wp_ = 0.101, *R*_exp_ = 0.060, *R*(*F* ^2^) = 0.03552, χ^2^ = 2.856
No. of parameters	95
No. of restraints	20
H-atom treatment	H atoms treated by a mixture of independent and constrained refinement
(Δ/σ)_max_	0.17

Computer programs: local program at 11BM, *GSAS* (Larson & Von Dreele, 2000 ▸), coordinates from an isotypic structure, *CrystalMaker* (Palmer, 2005 ▸), *publCIF* (Westrip, 2010 ▸).

## supplementary crystallographic information

### Crystal data

**Table:**

(NH_4_)_3_Al_2_(PO_4_)_3_	*V* = 1327.11 (2) Å^3^
*M**_r_* = 392.99	*Z* = 4
Orthorhombic, *P**n**a*2_1_	Synchrotron radiation, λ = 0.373811 Å
Hall symbol: P 2c -2n	µ = 0.12 mm^−^^1^
*a* = 8.98884 (6) Å	*T* = 100 K
*b* = 17.01605 (10) Å	white
*c* = 8.67653 (5) Å	cylinder, 0.70 × 0.70 mm

### Data collection

**Table:**

11BM_synchrotron diffractometer	Scan method: continuous
Specimen mounting: capillary	2θ_min_ = 2.45°, 2θ_max_ = 20°, 2θ_step_ = 0.001°
Data collection mode: transmission	

### Refinement

**Table:**

Least-squares matrix: full	Profile function: CW Profile function number 4 with 18 terms Pseudovoigt profile coefficients as parameterized in P. Thompson, D.E. Cox & J.B. Hastings (1987). J. Appl. Cryst.,20,79-83. Asymmetry correction of L.W. Finger, D.E. Cox & A. P. Jephcoat (1994). J. Appl. Cryst.,27,892-900. Microstrain broadening by P.W. Stephens, (1999). J. Appl. Cryst.,32,281-289. #1(GU) = 1.163 #2(GV) = -0.126 #3(GW) = 0.063 #4(GP) = 0.000 #5(LX) = 0.143 #6(ptec) = -0.01 #7(trns) = 0.00 #8(shft) = 0.0000 #9(sfec) = 0.00 #10(S/L) = 0.0011 #11(H/L) = 0.0011 #12(eta) = 0.7694 #13(S400 ) = 1.1E-01 #14(S040 ) = 2.8E-03 #15(S004 ) = 1.0E-01 #16(S220 ) = 1.3E-02 #17(S202 ) = -9.0E-03 #18(S022 ) = 6.9E-03 Peak tails are ignored where the intensity is below 0.0010 times the peak Aniso. broadening axis 1.0 0.0 0.0
*R*_p_ = 0.082	95 parameters
*R*_wp_ = 0.101	20 restraints
*R*_exp_ = 0.060	H atoms treated by a mixture of independent and constrained refinement
*R*(*F*^2^) = 0.03552	(Δ/σ)_max_ = 0.17
49495 data points	Background function: GSAS Background function number 1 with 11 terms. Shifted Chebyshev function of 1st kind 1: 111.086 2: -42.2923 3: 17.4011 4: -1.76183 5: -7.25556 6: 2.97020 7: 2.60010 8: -3.84672 9: 5.91765 10: -3.48127 11: 1.19076

### Fractional atomic coordinates and isotropic or equivalent isotropic displacement parameters (Å^2^)

**Table:**

	*x*	*y*	*z*	*U*_iso_*/*U*_eq_	
P1	0.1689 (3)	0.21458 (16)	−0.0138 (5)	0.0086 (3)*	
P2	0.3100 (3)	0.29952 (16)	0.4927 (5)	0.0086 (3)*	
P3	0.2594 (3)	0.5033 (2)	0.0861	0.0086 (3)*	
Al1	0.3631 (4)	0.33375 (18)	0.1361 (5)	0.0086 (3)*	
Al2	0.1295 (4)	0.17048 (19)	0.6462 (5)	0.0086 (3)*	
O1	0.2886 (7)	0.1552 (3)	0.0117 (8)	0.0086 (3)*	
O2	0.0384 (8)	0.2046 (3)	0.1014 (8)	0.0086 (3)*	
O3	0.2289 (7)	0.3006 (3)	0.0100 (8)	0.0086 (3)*	
O4	0.1017 (7)	0.2138 (4)	0.8252 (8)	0.0086 (3)*	
O5	0.1953 (7)	0.3602 (3)	0.5464 (8)	0.0086 (3)*	
O6	0.4625 (7)	0.3234 (3)	0.5425 (8)	0.0086 (3)*	
O7	0.2776 (8)	0.2165 (3)	0.5518 (7)	0.0086 (3)*	
O8	0.3087 (7)	0.2962 (4)	0.3126 (8)	0.0086 (3)*	
O9	0.1072 (5)	0.4838 (3)	0.1404 (8)	0.0086 (3)*	
O10	0.3228 (7)	0.5700 (4)	0.1747 (7)	0.0086 (3)*	
O11	0.2604 (6)	0.5218 (3)	0.9186 (6)	0.0086 (3)*	
O12	0.3716 (7)	0.4348 (3)	0.1188 (9)	0.0086 (3)*	
N1	0.0164 (8)	0.3978 (4)	0.8194 (9)	0.0086 (3)*	
H11	0.076 (5)	0.425 (3)	0.893 (4)	0.0086 (3)*	
H12	−0.058 (4)	0.368 (3)	0.871 (5)	0.0086 (3)*	
H13	0.077 (5)	0.364 (2)	0.760 (5)	0.0086 (3)*	
H14	−0.030 (5)	0.435 (2)	0.753 (5)	0.0086 (3)*	
N2	0.9630 (8)	0.3743 (4)	0.3168 (9)	0.0086 (3)*	
H21	1.036 (4)	0.363 (3)	0.393 (5)	0.0086 (3)*	
H22	0.915 (6)	0.3268 (16)	0.287 (6)	0.0086 (3)*	
H23	1.009 (5)	0.397 (3)	0.229 (4)	0.0086 (3)*	
H24	0.891 (5)	0.410 (3)	0.358 (6)	0.0086 (3)*	
N3	0.6786 (6)	0.4917 (4)	0.1118 (7)	0.0086 (3)*	
H31	0.755 (4)	0.530 (2)	0.095 (6)	0.0086 (3)*	
H32	0.668 (5)	0.483 (3)	0.2196 (14)	0.0086 (3)*	
H33	0.704 (5)	0.4438 (18)	0.062 (5)	0.0086 (3)*	
H34	0.587 (3)	0.511 (3)	0.071 (5)	0.0086 (3)*	

### Geometric parameters (Å, º)

**Table:**

P1—Al1	2.975 (5)	O3—P1	1.573 (6)
P1—Al2^i^	3.064 (5)	O3—Al1	1.724 (7)
P1—O1	1.493 (6)	O4—P1^iv^	1.522 (7)
P1—O2	1.550 (7)	O4—Al2	1.737 (7)
P1—O3	1.573 (6)	O5—P2	1.532 (6)
P1—O4^i^	1.522 (7)	O5—H21^viii^	1.953 (13)
P2—Al2	3.038 (4)	O5—H31^ix^	1.97 (3)
P2—O5	1.532 (6)	O6—P2	1.493 (7)
P2—O6	1.493 (7)	O6—Al2^iii^	1.753 (7)
P2—O7	1.532 (6)	O7—P2	1.532 (6)
P2—O8	1.563 (6)	O7—Al2	1.748 (7)
P3—Al1	3.062 (5)	O8—P2	1.563 (6)
P3—Al2^ii^	3.060 (5)	O8—Al1	1.730 (7)
P3—O9	1.484 (5)	O9—P3	1.484 (5)
P3—O10	1.485 (6)	O9—H14^x^	1.828 (10)
P3—O11^i^	1.487 (5)	O9—H23^viii^	1.88 (3)
P3—O12	1.567 (6)	O10—P3	1.485 (6)
Al1—P1	2.975 (5)	O10—Al2^ii^	1.780 (7)
Al1—P3	3.062 (5)	O11—P3^iv^	1.487 (5)
Al1—O2^iii^	1.732 (7)	O11—H24^ix^	1.868 (10)
Al1—O3	1.724 (7)	O11—H32^ix^	1.84 (2)
Al1—O8	1.730 (7)	O12—P3	1.567 (6)
Al1—O12	1.727 (6)	O12—Al1	1.727 (6)
Al2—P1^iv^	3.064 (5)	N1—H11	0.9526 (1)
Al2—P2	3.038 (4)	N1—H12	0.9526 (1)
Al2—P3^v^	3.060 (5)	N1—H13	0.9526 (1)
Al2—O4	1.737 (7)	N1—H14	0.9526
Al2—O6^vi^	1.753 (7)	N2—H21	0.9526 (1)
Al2—O7	1.748 (7)	N2—H22	0.9526 (1)
Al2—O10^v^	1.780 (7)	N2—H23	0.9526 (1)
O1—P1	1.493 (6)	N2—H24	0.9526 (1)
O1—H12^vii^	1.88 (2)	N3—H31	0.9526 (1)
O1—H33^vi^	1.897 (15)	N3—H32	0.9526 (1)
O2—P1	1.550 (7)	N3—H33	0.9526 (1)
O2—Al1^vi^	1.732 (7)	N3—H34	0.9526 (1)
			
O1—P1—O2	112.1 (4)	O6^vi^—Al2—O10^v^	109.6 (3)
O1—P1—O3	111.3 (4)	O7—Al2—O10^v^	108.1 (3)
O1—P1—O4^i^	114.6 (4)	P1—O2—Al1^vi^	147.3 (5)
O2—P1—O3	106.1 (4)	P1—O3—Al1	128.9 (4)
O2—P1—O4^i^	106.9 (4)	P1^iv^—O4—Al2	140.1 (4)
O3—P1—O4^i^	105.4 (4)	P2—O6—Al2^iii^	161.9 (5)
O5—P2—O6	110.3 (4)	P2—O7—Al2	135.6 (5)
O5—P2—O7	113.1 (4)	P2—O8—Al1	150.4 (4)
O5—P2—O8	108.9 (4)	P3—O10—Al2^ii^	139.0 (5)
O6—P2—O7	109.2 (4)	P3—O12—Al1	136.7 (5)
O6—P2—O8	107.8 (4)	H11—N1—H12	109.4713 (9)
O7—P2—O8	107.4 (4)	H11—N1—H13	109.4719 (6)
O9—P3—O10	111.1 (4)	H11—N1—H14	109.4706
O9—P3—O11^i^	111.3 (4)	H12—N1—H13	109.4715 (1)
O9—P3—O12	111.7 (4)	H12—N1—H14	109.4704
O10—P3—O11^i^	110.0 (4)	H13—N1—H14	109.4715 (5)
O10—P3—O12	103.2 (3)	H21—N2—H22	109.4716 (7)
O11^i^—P3—O12	109.3 (4)	H21—N2—H23	109.4710 (4)
O2^iii^—Al1—O3	113.8 (4)	H21—N2—H24	109.4716 (4)
O2^iii^—Al1—O8	105.8 (4)	H22—N2—H23	109.4707
O2^iii^—Al1—O12	108.6 (4)	H22—N2—H24	109.4717 (8)
O3—Al1—O8	104.1 (3)	H23—N2—H24	109.4706 (7)
O3—Al1—O12	107.6 (4)	H31—N3—H32	109.4709
O8—Al1—O12	117.2 (4)	H31—N3—H33	109.4710 (5)
O4—Al2—O6^vi^	108.1 (3)	H31—N3—H34	109.4709 (1)
O4—Al2—O7	109.8 (3)	H32—N3—H33	109.4717
O4—Al2—O10^v^	108.5 (4)	H32—N3—H34	109.472
O6^vi^—Al2—O7	112.6 (4)	H33—N3—H34	109.4709

Symmetry codes: (i) *x*, *y*, *z*−1; (ii) −*x*+1/2, *y*+1/2, *z*−1/2; (iii) *x*+1/2, −*y*+1/2, *z*; (iv) *x*, *y*, *z*+1; (v) −*x*+1/2, *y*−1/2, *z*+1/2; (vi) *x*−1/2, −*y*+1/2, *z*; (vii) *x*+1/2, −*y*+1/2, *z*−1; (viii) *x*−1, *y*, *z*; (ix) −*x*+1, −*y*+1, *z*+1/2; (x) −*x*, −*y*+1, *z*−1/2.

### Hydrogen-bond geometry (Å, º)

**Table:**

*D*—H···*A*	*D*—H	H···*A*	*D*···*A*	*D*—H···*A*
N1—H11···O9^iv^	0.95 (4)	2.39 (4)	3.250 (10)	151 (3)
N1—H11···O11	0.95 (4)	2.35 (5)	3.163 (8)	143 (3)
N1—H12···O1^xi^	0.95 (4)	1.88 (4)	2.791 (10)	159 (3)
N1—H13···O5	0.95 (4)	2.14 (4)	2.934 (10)	141 (3)
N1—H14···O9^xii^	0.95 (4)	1.83 (4)	2.776 (9)	173 (3)
N2—H21···O5^xiii^	0.95 (4)	1.96 (4)	2.896 (10)	170 (4)
N2—H22···O8^iii^	0.95 (4)	2.31 (3)	3.216 (10)	158 (4)
N2—H23···O9^xiii^	0.95 (4)	1.89 (5)	2.738 (9)	148 (4)
N2—H24···O11^xiv^	0.96 (4)	1.86 (4)	2.818 (9)	174 (5)
N3—H31···O5^xiv^	0.96 (4)	1.97 (4)	2.821 (9)	147 (3)
N3—H32···O11^xiv^	0.952 (15)	1.85 (2)	2.728 (8)	153 (4)
N3—H33···O1^iii^	0.95 (3)	1.90 (3)	2.823 (9)	164 (4)
N3—H34···O12	0.96 (3)	2.37 (4)	2.925 (8)	117 (4)

Symmetry codes: (iii) *x*+1/2, −*y*+1/2, *z*; (iv) *x*, *y*, *z*+1; (xi) *x*−1/2, −*y*+1/2, *z*+1; (xii) −*x*, −*y*+1, *z*+1/2; (xiii) *x*+1, *y*, *z*; (xiv) −*x*+1, −*y*+1, *z*−1/2.

## References

[bb1] Boughzala, H., Driss, A. & Jouini, T. (1997). *Acta Cryst.* C**53**, 3–5.

[bb2] Byrne, P. J., Warren, J. E., Morris, R. E. & Ashbrook, S. E. (2009). *Solid State Sci.* **11**, 1001–1006.

[bb3] Cooper, E. R., Andrews, C. D., Wheatley, P. S., Webb, P. B., Wormald, P. & Morris, R. E. (2004). *Nature*, **430**, 1012–1016. 10.1038/nature02860 15329717

[bb4] Das, S. K., Bhunia, M. K. & Bhaumik, A. (2012). *Microporous Mesoporous Mater.* **155**, 258–264.

[bb10] Devi, R. N. & Vidyasagar, K. (2000). *Inorg. Chem.* **39**, 2391–2396. 10.1021/ic991395u 12526501

[bb5] Larson, A. C. & Von Dreele, R. B. (2000). *General Structure Analysis System* (*GSAS*). Report LAUR, 86–748 Los Alamos National Laboratory, New Mexico, USA.

[bb6] Li, Y., Yu, J. & Xu, R. (2019). *URL of AlPO database (freely accessible)*: http://mezeopor.jlu.edu.cn/alpo/alpo.jsp

[bb7] Ma, H., Tian, Z., Xu, R., Wang, B., Wei, Y., Wang, L., Xu, Y., Zhang, W. & Lin, L. (2008). *J. Am. Chem. Soc.* **130**, 8120–8121. 10.1021/ja802207p 18529056

[bb8] Medina, M. E., Iglesias, M., Gutiérrez-Puebla, E. & Monge, M. A. (2004). *J. Mater. Chem.* **14**, 845–850.

[bb9] Morris, R. E. (2009). *Chem. Commun.* pp. 2990–2998. 10.1039/b902611h 19462065

[bb11] Oliver, S., Kuperman, A., Lough, A. & Ozin, G. A. (1996). *Chem. Commun.* pp. 1761–1762.

[bb12] Palmer, D. (2005). *CrystalMaker*. CrystalMaker Software Ltd, Yarnton, England.

[bb13] Parnham, E. & Morris, R. E. (2007). *Acc. Chem. Res.* **40**, 1005–1013. 10.1021/ar700025k 17580979

[bb14] Richardson, J. W. & Vogt, E. T. C. (1992). *Zeolites*, **12**, 13–19.

[bb15] Shannon, R. D. (1976). *Acta Cryst.* A**32**, 751–767.

[bb16] Sidey, V. (2016). *Acta Cryst.* B**72**, 626–633. 10.1107/S2052520616008064 27484382

[bb17] Stöger, B. & Weil, M. (2012). *Acta Cryst.* E**68**, i15. 10.1107/S1600536812000438 PMC3274838 22346791

[bb18] Vaughan, D. E. W., Yennawar, H. P. & Perrotta, A. J. (2012). *Microporous Mesoporous Mater.* **153**, 18–23.

[bb19] Wei, Y., Marler, B., Zhang, L., Tian, Z., Graetsch, H. & Gies, H. (2012). *Dalton Trans.* **41**, 12408–12415. 10.1039/c2dt31150j 22940750

[bb20] Westrip, S. P. (2010). *J. Appl. Cryst.* **43**, 920–925.

[bb21] Wilson, S. T. (2007). *Introduction to Zeolite Science and Practice*, 3rd ed, edited by J. Čejka, H. van Bekkum, A. Corma, & F. Schüth, ch. 4, pp. 105–135. Amsterdam: Elsevier.

[bb22] Wilson, S. T., Lok, B. M., Messina, C. A., Cannan, T. R. & Flanigen, E. M. (1982). *J. Am. Chem. Soc.* **104**, 1146–1147.

[bb23] Xing, H., Li, J., Yan, W., Chen, P., Jin, Z., Yu, J., Dai, S. & Xu, R. (2008). *Chem. Mater.* **20**, 4179–4181.

[bb24] Xing, H., Li, Y., Su, T., Xu, J., Yang, W., Zhu, E., Yu, J. & Xu, R. (2010). *Dalton Trans.* **39**, 1713–1715. 10.1039/b919933k 20449410

[bb25] Yu, J. & Xu, R. (2006). *Chem. Soc. Rev.* **35**, 593–604.

